# Advancing Access to Diagnostic Tools Essential for Universal Health Coverage and Antimicrobial Resistance Prevention: An Overview of Trials in Sub-Saharan Africa

**DOI:** 10.1093/cid/ciad326

**Published:** 2023-07-25

**Authors:** Piero Olliaro, Juvenal Nkeramahame, Olawale Salami, Catrin E Moore, Philip Horgan, Rita Baiden, Vida Kukula, Alexander Adjei, James Kapisi, Heidi Hopkins, David Kaawa-Mafigiri, Deborah Ekusai-Sebatta, Elizeus Rutebemberwa, Freddy Eric Kitutu, Halidou Tinto, François Kiemde, Adélaïde Compaoré, Daniel Valia, Sabine Dittrich, the ADIP study group, Phyllis Awor, Deborah Ekusai-Sebatta, Heidi Hopkins, David Kaawa–Mafigiri, James Kapisi, Freddy Eric Kitutu, Elizeus Rutebemberwa, Asadu Sserwanga, Alexander Adjei, Emmanuel Arthur, Elizabeth Awini, Rita Baiden, Vida Kukula, Clement Tetteh Narh, Gabriel Odonkor, Selase Odopey, John Williams, Adélaïde Compaoré, François Kiemde, Halidou Tinto, Daniel Valia

**Affiliations:** International Severe Acute Respiratory and Emerging Infection Consortium, Pandemic Sciences Institute, University of Oxford, Oxford, United Kingdom; FIND, Geneva, Switzerland; FIND, Geneva, Switzerland; FIND, Geneva, Switzerland; Nuffield Department of Medicine, Big Data Institute, University of Oxford, Oxford, United Kingdom; Centre for Neonatal and Paediatric Infection, Institute for Infection and Immunity, St George's University of London, London, United Kingdom; FIND, Geneva, Switzerland; Nuffield Department of Medicine, Big Data Institute, University of Oxford, Oxford, United Kingdom; Evidence & Impact Oxford, Oxford, United Kingdom; INDEPTH-Network, Accra, Ghana; Dodowa Health Research Centre, Dodowa, Ghana; Dodowa Health Research Centre, Dodowa, Ghana; Infectious Diseases Research Collaboration, Kampala, Uganda; London School of Hygiene & Tropical Medicine, London, United Kingdom; Social Work and Social Administration, Makerere University, Kampala, Uganda; Infectious Diseases Research Collaboration, Kampala, Uganda; Department of Health Policy and Planning, Makerere University School of Public Health, Kampala, Uganda; Department of Pharmacy, Makerere University School of Health Sciences, Kampala, Uganda; Clinical Research Unit of Nanoro, Institut de Recherche en Sciences de La Santé, Nanoro, Burkina Faso; Clinical Research Unit of Nanoro, Institut de Recherche en Sciences de La Santé, Nanoro, Burkina Faso; Clinical Research Unit of Nanoro, Institut de Recherche en Sciences de La Santé, Nanoro, Burkina Faso; Clinical Research Unit of Nanoro, Institut de Recherche en Sciences de La Santé, Nanoro, Burkina Faso; FIND, Geneva, Switzerland; Center for Tropical Medicine and Global Health, Nuffield Department of Medicine, University of Oxford, Oxford, United Kingdom; Deggendorf Institute of Technology, European Campus Rottal Inn, Pfarrkirchen, Germany

## Abstract

We introduce the Antimicrobial Resistance Diagnostic Use Accelerator program, and the articles in this Supplement, which cover the program in 3 sub-Saharan Africa countries.

## BACKGROUND

Acute febrile illness (AFI) is one of the main reasons for consultation at first-level health facilities such as peripheral health clinics and hospital outpatient departments, but the causative agents are often largely unknown [1].

AFIs pose a two-fold challenge: For healthcare providers, the immediate question of managing the individual patient; and to health systems, the longer-term consequences on health and the economy of inappropriate case management including antimicrobial resistance (AMR). Both phenomena are multi-factorial, but they have a common cause: limited access to, or absence of appropriate diagnostic tools, especially point-of-care (POC) tests to orient the treatment choice towards an antibiotic or other treatment. These challenges are faced by healthcare practitioners worldwide, but the problem is particularly serious in low-resource countries.

Information on the incidence and causes of AFI in different parts of the world is fragmented. Data vary greatly even across a single continent, for example, sub-Saharan Africa [2, 3]. Estimates suggest that children experience on average 6 fever episodes each year before they reach 5 years of age [4]. The combination of POC rapid diagnostic tests (RDTs) to diagnose malaria and a trend toward a decline in malaria transmission in some areas has exposed the high burden of non-malarial AFIs and mixed infections, which are often treated empirically with antibiotics [5]. According to the World Health Organization (WHO) informal consultation on fever management in peripheral healthcare settings, “most (50%–75%) febrile episodes in children under 5 years of age presenting at outpatient clinics are associated with acute respiratory infections” [1, 6], which are predominantly being caused by viral pathogens [7]. Similarly, a recent literature review found that up to 60% of children presenting with fever at first-level health facilities have self-limiting arboviral or viral upper respiratory tract infections [8]. Using data from 21 sub-Saharan Africa countries over a decade (2006–2016), estimates show that ∼38% of fevers in children under 5 years are attributable to malaria [9].

Lacking practical alternatives, healthcare providers often revert to “just-in-case” antibiotics, which is considered one of the major contributing factors to increasing AMR [10]. According to the most recent modelled estimates of AMR burden, 1.27 million (95% uncertainty interval [UI] 0.91–1.71) deaths were attributable to bacterial AMR [11]. The region with highest risk was western sub-Saharan Africa with 27.3 deaths (95% UI: 20.9–35.3) per 100 000 people [11]. However, providing accurate estimates of AMR related outcomes is challenging, given the scarcity of data. The combination of a largely unexplored spectrum of causative agents for infection even using laboratory-based tests, the limited availability of appropriate diagnostic tools, the availability of antibiotics, and the low number of POC tests that can be applied in routine patient care—either because they do not exist or cannot be afforded—restricts the options for improvements at first-level health facilities.

FIND (https://www.finddx.org/) seeks to ensure equitable access to reliable diagnosis around the world, we collaborate with countries and developers to spur diagnostic innovation and make testing an integral part of sustainable, resilient health systems. The AMR Diagnostic Use Accelerator program (https://www.finddx.org/what-we-do/projects/amr-dx-use-accelerator/) was set up to identify practical solutions that can be applied today, using commercially available diagnostic tools that can be used near-patient and supported by a decision-making aid. Here we report the results of the Advancing Access to Diagnostic Innovation essential for Universal Health Coverage and AMR Prevention (ADIP) trials in 3 sub-Saharan Africa countries.

The question to be answered: the PICO (Population, Intervention, Control, Outcomes) framework.

The aim of the ADIP studies was to evaluate if:

- by combining available POC diagnostic tests in diagnostic aids/algorithms, and training and communication for patients and caregivers (the Intervention);
- we could improve the case management of acute, non-severe febrile illnesses and the targeted use of antibiotics, thereby reducing antibiotic prescriptions (Outcomes);
- in patients with undifferentiated, non-severe acute febrile illness presenting to outpatient clinics and/or peripheral health centers in low-resource countries (Population);
- compared with current clinical practice (Control).

The primary study outcomes (antibiotic prescriptions and clinical outcome) were evaluated on Day 0 and at Day 7 of follow-up, respectively. The primary endpoint was whether an antibiotic was prescribed at Day 0 and whether the patient was alive and with symptoms resolved at Day 7, respectively. The antibiotic prescription rate refers to the number of prescriptions from the prescribers which included an antibiotic. As there was only 1 prescription per patient, this is the same as the number of patients who received a prescription that included an antibiotic.

### The Approach: Combining Clinical Decision Making and Social Science

**Figure 1.**
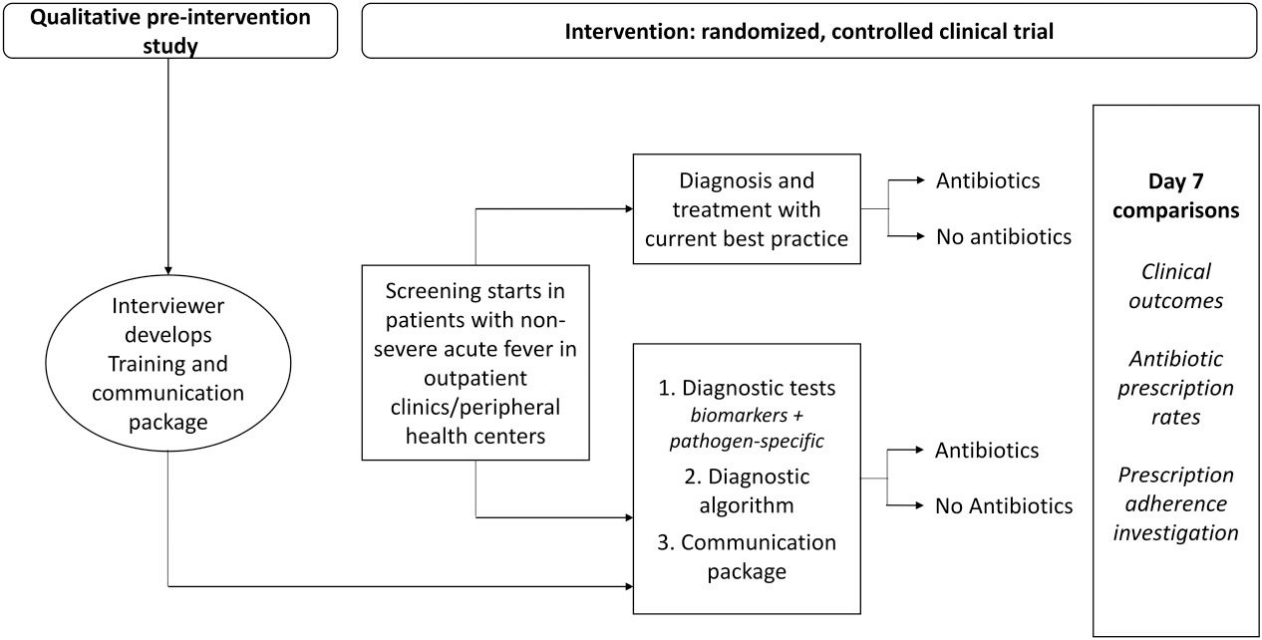
Qualitative pre-intervention and intervention study design.

The likelihood of achieving these outcomes depends on the adequacy of the tools made available (eg, diagnostic test performance, usefulness of diagnostic algorithms) as well as the acceptability of, and adherence to, the interventions by both prescribers and users. For these reasons we developed an approach combining both clinical and social science methodology. The protocol, which included study sites in Africa and Asia, was registered on clinicaltrials.gov (NCT04081051) and published [12]. Here we report the results of the studies conducted at facilities in 3 sub-Saharan African countries: Burkina Faso, Ghana, and Uganda. The study design (Figure 1) included: (1) a qualitative pre-intervention study to understand the patients’ behavior toward prescribed medicine, and prescribers’ communication of adherence messages; and (2) a randomized comparative clinical trial with a qualitative and behavioral component.

### Overview of Clinical Study Sites

The studies covered a range of typical settings in sub-Saharan Africa: hospital outpatient departments in Level I and II healthcare facilities in rural Burkina Faso (equivalent to primary health centers; an urban/peri-urban area of Ghana; and health centers IV in rural Uganda (equivalent to subdistrict hospitals; Table 1) [13, 14]. Sites had different levels of malaria endemicity and varying transmission patterns. The sites also employed health workers with wide-ranging clinical diagnostic training, from nurses in rural Burkina Faso, through clinical officers in Uganda, to doctors/physician assistants in Ghana.

**Table 1.** Trial Site's Main Characteristics

Country	Setting and Sites	Expected Antibiotic Prescription Rate (Based on Historic Prescribing)	Malaria Endemicity and Transmission Patterns	Projected Total Sample Size Over 12 m^a^	Study Population
Burkina Faso	*Rural* Pella and Temnaore health centers	77%	Seasonal: high transmission: June to July and October to November	1718	Children and adolescents (6 m to <18 y)
Ghana	*Urban, semi-urban* Shai-Osudoku District hospital, St. Andrew's Catholic hospital, Pramram District hospital and Ningo health center	43%	Seasonal: July to October^b^	2766	Children and adolescents (6 m to <18 y)
Uganda	*Rural* Aduku level IV health center, Nagongera level IV health center Kihihi level IV health center	73%	High, low, and moderate malaria transmission depending on the site^c^	2400	Children (>1 y), Adolescents, adults

Both arms, including losses to follow-up.

Malaria season typically runs March to October but varies between ecological seasons. In the Shai-Osudoku area where the study was conducted, the seasons run from May to June and September to November.

Please see the Uganda clinical trial manuscript later in the Supplement for further information (Kapisi J, et al, *CID* Supplement 2023).

### Qualitative Pre-intervention Study

It was assumed that the benefits to patients of using diagnostics will not be realized if patients’ adherence to the resulting prescriptions is weak. We therefore included a training and communication package in the intervention package, designed based on pre-intervention research findings, in support of prescription adherence.

The pre-intervention, qualitative research study was conducted to investigate the social, economic, and cultural factors that support or hinder patient's adherence to prescriptions and the communication of adherence messages from healthcare workers (Figure 1). Tailored to local context, and building on the Capability, Opportunity and Motivation model of Behaviors (COM-B) [15], and Theoretical Domains Framework (TDF) [16], in-depth-interviews and focus group discussions were conducted with patients/caregivers and prescribers to explore the topic. Both frameworks categorize drivers of behaviors and were used to inform the design of research topic guides and to support the exploration of behavioral factors that support or hinder patients’ adherence to prescriptions.

Country context-specific training and communication (T&C) intervention packages were developed independently of the other sites using simplified language to respond to the specific behavioral drivers (ie, the supporting or hindering factors identified in the pre-intervention study) to support the patient's adherence to prescription. The T&C packages were pretested for clarity, as well as training for healthcare workers such that healthcare workers could effectively communicate messages. Each T&C package consisted of a set of communication messages which were presented to patients in local languages at the point of prescribing to support patient adherence to prescription.

### Choice of Suitable Point-of Care Rapid Diagnostic Tests

The availability of RDTs in resource-constrained peripheral health centers is a potential game-changer for the diagnosis and management of common febrile illnesses. Malaria gives a striking example: the rollout of simple RDTs for malaria in endemic countries, alongside supporting measures, changed the behavior and treating practices of healthcare workers when WHO policy changed to “test and treat.” At the same time, the diagnosis of many other infections causing acute undifferentiated febrile illness and respiratory tract infections, which are among the most common reasons for attending an outpatient clinic, remains elusive [17].

Ideally, simple tools would be used that can detect both viruses and specific bacteria to direct antibiotic treatment. As such, healthcare workers would ideally have a panel of RDTs covering the spectrum of the prevalent pathogens. Hence, we aimed for RDTs that could have the potential to modify prescribing habits.

RDT selection was based on the following criteria: RDTs had to (1) be currently available on the international market and have European CE mark or US Food and Drug Administration (FDA) approval; (2) be fit to be used in outpatient clinics in LMICs by a primary healthcare worker, with or without the guidance of a trained laboratory person; (3) be able to identify infections likely to be prevalent at the study sites or aid the differentiation between viral and bacterial infections; and (4) be approved for use in the country of the study site for either research or diagnostic purposes.

Following these criteria, we selected seven pathogen-specific tests for: malaria, group A Streptococcus, influenza virus, respiratory syncytial virus (RSV) for children aged <2 years, *Streptococcus pneumoniae*, dengue virus, and *Salmonella enterica* serovar Typhi (enteric fever) and 3 general biomarkers of acute infection (white blood cell differentiation, C-reactive protein [CRP], and urine dipstick for nitrites and leucocyte esterase; Table 2).

**Table 2.** Tests Used and Their Reported Performance

Pathogen	Type of Test	Test Name and Manufacturer	Test Performance by Manufacturer and Independent Studies
*Streptococcus pyogenes* (Group A streptococci)	Lateral flow RDT: detects Group A streptococcal antigen from throat swabs	OSOM Strep A, Sekisui Diagnostics	Sensitivity 96%; specificity 98% (vs culture; from Manufacturer) [18] Sensitivity 98%; specificity 99% (vs culture) [19] Sensitivity 86% (83.3–87.6); specificity 95% (94.5–96.2) [20]
*Streptococcus pneumoniae*	Lateral flow RDT: detects *S. pneumoniae* antigen in the urine of patients with pneumococcal pneumonia	BinaxNOW^™^ *Streptococcus pneumoniae* antigen card, Abbott/Alere	Sensitivity 86%; specificity 94% (from Manufacturer; urine test) [21] Sensitivity 74%; specificity 97% (from retrospective data) [22]
Influenza virus	Lateral flow RDT: detects influenza virus type A, type B and A (H1N1) pandemic antigens directly from nasal/throat/nasopharyngeal swab or nasal/nasopharyngeal aspirate	SD Bioline influenza Ag A/B/A(H1N1) PANDEMIC, Abbott/Alere	Sensitivity/specificity not available not available from Manufacturer [23] Sensitivity 56%; specificity 100% (vs RT-PCR) [24]
RSV	Lateral flow RDT: detects respiratory syncytial virus fusion protein antigen in nasal wash and nasopharyngeal swab specimens	Alere BinaxNOW® RSV Card, Abbott	Sensitivity 93%; specificity 93% (from Manufacturer; prospective NP swab); sensitivity 89%; specificity 100% (retrospective nasal wash) [25] Sensitivity 81%; specificity 93% (vs culture) [26]
*Salmonella enterica* serovar Typhi	Lateral flow RDT: detects *Salmonella typhi*-specific IgM from serum or whole blood	Test-it™ Typhoid IgM lateral flow assay, Life Assay Diagnostics Pty Ltd	Sensitivity/specificity by Manufacturer (not available) [27] Sensitivity 69% (95% CI: 59%–78%) for age >1 y old; specificity 90% (95% CI: 78%–93%) [28]
Dengue^a^	Immunochromatographic assay	SD Bioline Dengue duo (NS1 Ag/IgG/IgM)	Sensitivity NS1 92%, IgM/IgG 94% (vs RT-PCR); specificity NS1 98%, IgG/IgM 96% (vs ELISA; From Manufacturer) [29] Sensitivity 89%; specificity 100% (vs0 ELISA) [30]
*Plasmodium* sp. (malaria)	Lateral flow RDT as per national guidelines	SD Bioline Malaria Ag P.F/PAN, Abbott	Sensitivity 100% (*Pf*), 95%(non-*Pf*); specificity 99% (from Manufacturer) [31] Sensitivity 99% (*Pf*), 93% (non-*Pf*); specificity, 98% (*Pf*), and 100%, non-*Pf*) [32]
CRP	Immunoassay for the quantitative measurement of CRP level in human serum, plasma and whole blood	Standard F CRP, SD Biosensor	Coefficient of variation 7.6%–8.1% (CRP; from Manufacturer) [33]
White blood cell differentiation	Five-part differentiation of white blood cell lines	HemoCue® WBC DIFF System, Hemocue	Measuring range: 0.3–30.0 × 10^9^/L (From Manufacturer) [34, 35] Reliable comparability in the range of 0.4–30.0 × 0^9^/L (vs calibrated reference blood cell analyzer) [36]
Urine tests	Urine dipstick test for urinary blood, bilirubin, ketones, pH, urobilinogen, protein, nitrites, leucocyte esterase and specific gravity	Multistix SG-10, Siemens SD Urocolor^TM^ 10, Abbott	*Multistix SG-10, Siemens* Sensitivity 88% (visual reading) 97% (instrument reading); specificity 93% (vs culture; from Manufacturer) [37] Sensitivity nitrite, leukocyte esterase, blood and protein 97% Specificity for nitrite, leukocyte esterase and blood 97% (94.2–98.6) (vs culture) [38] *SD Urocolor TM 10, Abbott* Sensitivity/specificity by Manufacturer (not available)

Abbreviations: CI, confidence interval; CRP, C-reactive protein; ELISA, enzyme-linked immunosorbent assay; IgG, immunoglobulin G; IgM, immunoglobulin M; NP, nasopharyngeal; *Pf*, *P. falciparum*; RDT, rapid diagnostic test; RSV, respiratory syncytial virus; RT-PCR, reverse transcription polymerase chain reaction; WBC, white blood cell.

Dengue tests were not performed in Uganda and Ghana.

This test selection, however, has limitations. First, they may not cover the spectrum of infections among the study population, which was not known and could vary, both from country to country and seasonally. Second, the number of infections that can be detected with an RDT is limited, and each test is standalone; therefore, several tests may need to be performed for each patient, which is an important factor in young children where samples can be difficult to collect. Third, test performance is not always well established, also given the lack of gold standards, which means that the predictive value of a positive or negative test may be questionable especially in the presence of varying disease prevalence. Fourth, tests results do not necessarily conclusively inform treatment choices. For instance, a positive test for a viral infection does not exclude a potential concomitant bacterial infection; or the detection of *Streptococcus pneumoniae* or group A *Streptococcus* from an upper respiratory sample in a child does not imply an aetiologic role in a concurrent respiratory illness, given the frequency of asymptomatic colonization with both organisms in early childhood. Specifically, the BinaxNOW urinary pneumococcal antigen test may not be of much utility in discriminating between children with and without pneumococcal LRTI due to nasopharyngeal carriage of *Streptococcus pneumoniae*, cross-reactivity with antigens from other colonizing bacteria, such as *Streptococcus mitis,* and from pneumococcal vaccination [39–41]. All these are factors prevalent in our study settings and may hinder the utility of the test.

### Randomized Comparative Clinical Trial Design

The primary study objectives were to assess the impact of diagnostic tools, clinical algorithms, and T&C packages on antibiotic prescriptions and clinical outcomes in patients presenting at outpatient clinics with acute febrile illness, compared with routine clinical practice.

Secondary study objectives were to assess adherence to the diagnostic algorithm by healthcare workers and adherence to prescriptions by patients/caregivers, as well as to evaluate the safety outcomes of these practices.

To be eligible, participants (children and young people in Burkina Faso and Ghana, all ages above 1 year in Uganda; Table 1) of both sexes, had to present with acute fever (ie, a temperature of >37.5°C or a history of fever within the last 7 days) either with no focus or with suspected respiratory tract infection, but lacking symptoms and signs of severe illness that required hospital admission or referral as assessed by the study healthcare workers. Participants had to provide informed consent/assent to provide blood and other samples, to adhere to study procedures such as taking medicines prescribed, and consent to return for a follow-up visit at the health facility on Day 7 (±2 days).

### Clinic Process Flow

**Figure 2.**
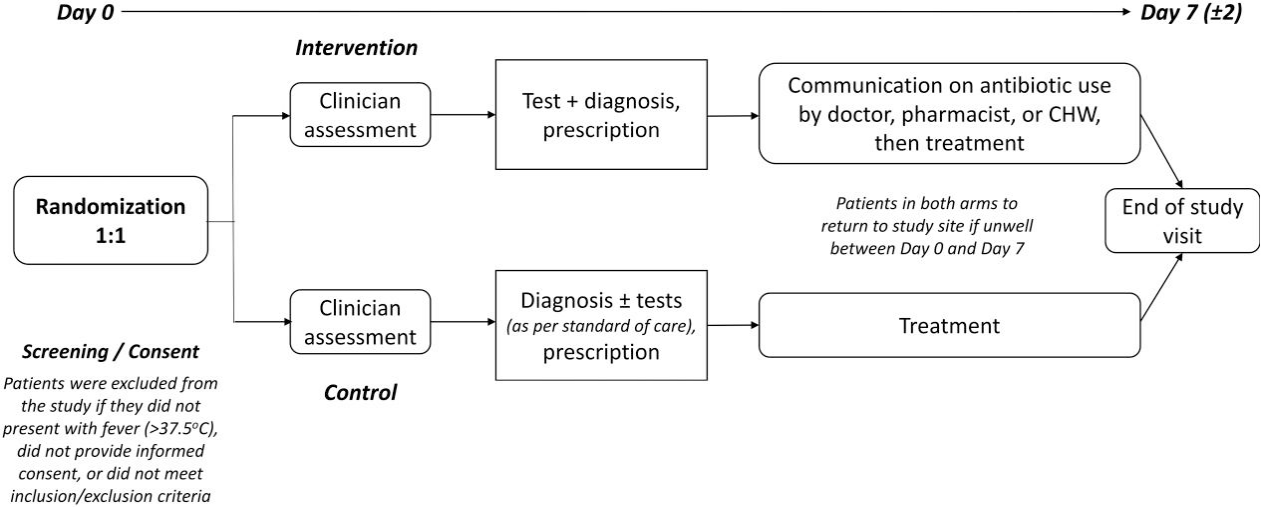
Clinical process flow after screening and informed consent. Site-specific adaptations will be detailed in individual clinical publications. Abbreviation: CHW, community health worker.

The participant flow is shown in Figure 2. Patients presenting at the clinic were pre-screened for fever. Participants who met the study eligibility criteria and who consented to participate in the study were randomized to the control or intervention arm of the study using randomization block sizes of 64, 96, and 128 in a 1:1 allocation ratio. Participants in both arms were seen by healthcare workers who collected histories and conducted clinical examinations.

**Figure 3.**
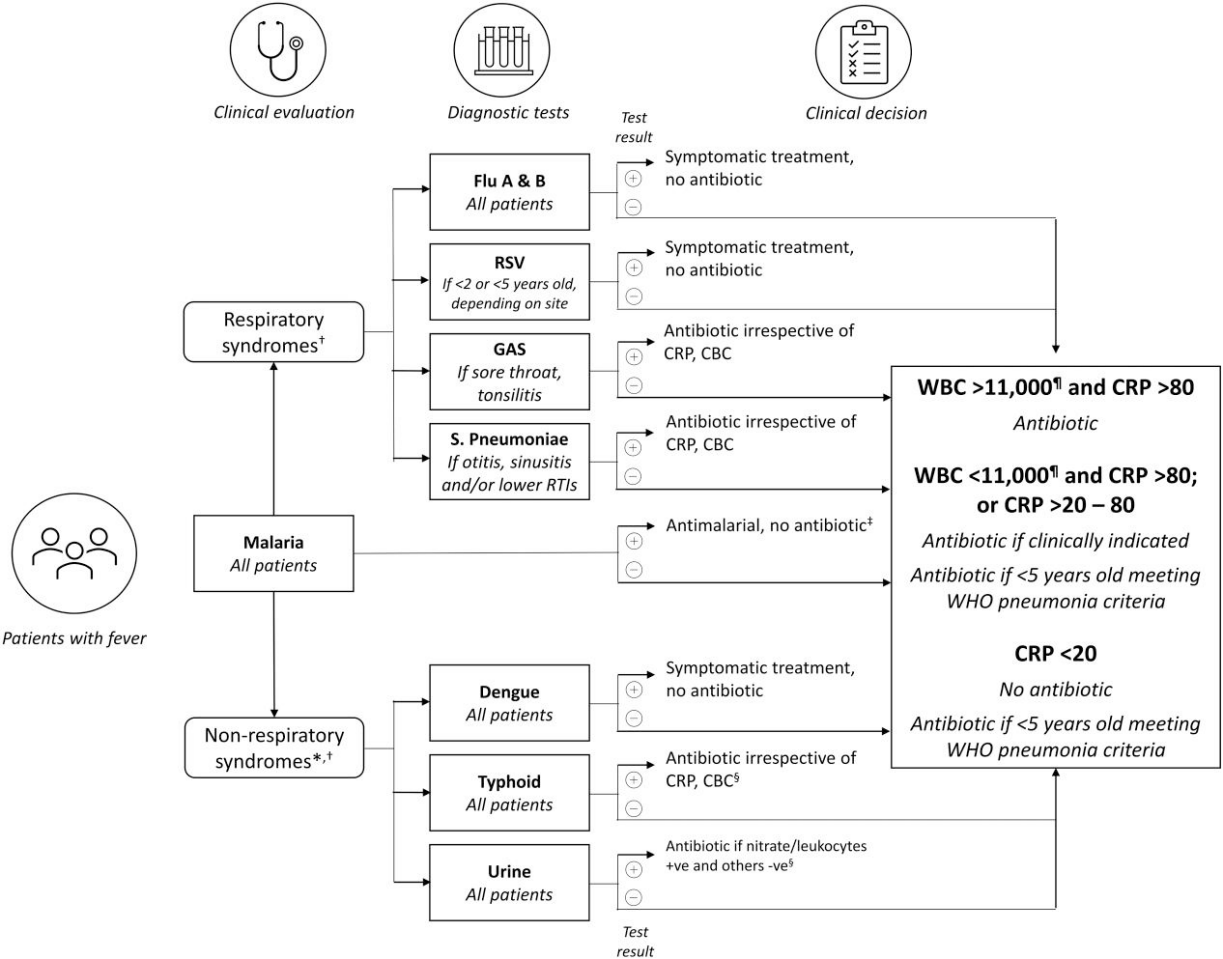
Fever clinical diagnostic algorithm—pathogen-specific tests. *Diagnostic panel depending on local endemicity; †Choice of tests at the discretion of local health practitioners; ‡Unless a concomitant bacterial pathogen identified; §Start treatment followed by culture if needed; ¶And neutrophils >75% if WBC >11 000 and/or neutrophils >75% if WBC <11 000. Abbreviations: CBC, complete blood count; CRP, C-reactive protein (mg/L); GAS, group A streptococci; RSV, respiratory syncytial virus; WBC, white blood cell count (per μL); WHO, World Health Organization.

Participants in the control arm followed routine diagnostic testing and treatment procedures at each site. Those in the intervention arm were first interviewed about their behaviors in relation to the use of antibiotics before being seen by a healthcare worker. Depending on the clinical presentation, the treating healthcare worker made a provisional diagnosis (respiratory/non-respiratory) and decided on which diagnostic tests were appropriate as guided by the diagnostic algorithm (Figure 3). All participants in both arms underwent a malaria test as per national guidelines. In the intervention arm, the decision to prescribe an antibiotic was based on the results of the tests and the algorithm. In the control arm, prescriptions were based on existing practice. Before leaving the clinic, participants in the intervention arm (only) also received additional personalized information based on the T&C guide to influence adherence to prescription (for antibiotic and non-antibiotic prescriptions).

All participants in both the intervention and control arms were asked to return on Day 7 (±2) to reassess their health status and prescription adherence.

### Fever Diagnostic Algorithm

In the intervention arm, the healthcare worker could decide which tests to apply, and based on test results, apply a pre-specified diagnostic algorithm (Figure 3) to determine whether to use an antibiotic to treat the condition.

In the control arm, the decision was based on the available standard of care tests (malaria RDT and any others, which may be variably available across countries and at different levels of the health system within the same country) and the healthcare worker's clinical judgement.

### Nested Qualitative and Behavioral Component

In addition to the pre-intervention qualitative study conducted to develop the T&C packages, further qualitative steps were nested into the clinical trial.

Dedicated social scientists conducted short interviews with intervention arm participants, to understand their usual behaviors in relation to prescriptions, prior to receiving the prescription on Day 0. To increase the effectiveness of the T&C messages, the prescriber personalized the messages in the T&C intervention for each participant based on this information.

Social scientists conducted qualitative semi-structured interviews with all patients or guardians in the intervention arm on Day 7, and a small portion of control arm patients, complementing clinical assessment. The interviews explored the participants’ behaviors and adherence to the prescription (defined as obtaining the prescribed medicines and taking them as per the prescription instructions of dosage, frequency, and duration), in addition to views on the T&C messages and future intentions to request antibiotics. Quantitative questions on adherence in the case report form were completed based on interview responses, providing an alternative to the self-reporting or pill count methods which were also used in the clinical follow-up assessment.

In each country, a sample of Day 7 interviews were analyzed using content analysis to better understand behaviors and the contextual factors that may have hindered or supported the prescription adherence in the trial.

In a final step in some sites, the Behavior Change Wheel framework [15] was used to identify recommendations to support adherence to prescriptions in the future. The Behavior Change Wheel process supports the design of interventions based on the categorization of behavioral drivers using the COM-B and TDF behavior frameworks.

### Uptake of Diagnostics

Furthermore, in a separate strand of work, social scientists conducted in-depth interviews with healthcare workers in the study clinics (both those from the study and the wider clinic staff) with responsibility for use of diagnostics, algorithms, and associated prescribing. The interviews explored the behavioral factors that hinder or support the uptake of available diagnostics, algorithms, and adherence to test results, both within the study and in general practice.

### Sample Size Considerations

The sample size was calculated individually for each country to be able to detect a 30% relative difference in the rate of antibiotic prescription between intervention and control arm, with a 95% confidence interval of ±5% on the estimate of the prescription rate in the intervention arm. The sample size calculations were also powered to have an 80% probability of observing a confidence interval of ±5% or less on the estimates in each control arm. The baseline prescription rates considered for the calculations were Burkina Faso: 77%; Ghana: 43%; and Uganda: 73%. The overall sample size was adjusted to account for potential loss to follow-up by increasing it by a factor of 10%.

### Ethical and Regulatory Considerations

The overarching protocol was reviewed and approved by the Human Research Ethics Committee of the University of Oxford, United Kingdom. In addition, country-specific protocols were reviewed and approved by relevant regulatory authorities and national and/or institutional ethics committees in all participating countries.

Written informed consent was provided by all participating adults and official caregivers of participating children.

### Site Preparation for Clinical Trial Initiation

Site preparatory activities were conducted in 4 parts:

1. *Site selection process*: A questionnaire was developed to evaluate sites’ capacity to recruit patients with acute febrile illnesses and their experience in the conduct of clinical trials. Sites were then selected based on the scoring of the received responses.
2. *Investigator meeting and collaborative protocol development*: Each applicant team prepared their own proposal in preparation for the site selection process. A combined study protocol was then developed by all the participating Investigators, working with the study team at FIND, University of Oxford, and partners from the Special Program for Research and Training in Tropical Diseases (WHO-TDR). The overarching protocol was adapted to develop country-specific protocols tailored to differences in the local epidemiology of acute febrile illnesses.
3. *Pre-study site visits*: The core team from FIND and the University of Oxford conducted an on-site evaluation of trial sites in Burkina Faso, Ghana, and Uganda to validate their capacity to enroll study participants and conduct the trial efficiently. In addition, emerging risks to trial implementation were identified and mitigation measures were agreed.
4. *Remote clinical trial site initiation*: With coronavirus disease 2019 (COVID-19)-associated travel bans imposed on several countries in early 2020, including the study countries, travel to trial sites for site initiation became impossible or extremely complicated. Therefore, the study team developed an operational model of a remote clinical trial site initiation visit, with measures in place to ensure the quality of testing and standardization of all diagnostic test processes as outlined in the study protocol, together with an evaluation of the competency of the diagnostic team. Furthermore, the remote study initiation process ensured that the workflow of all protocol-related procedures and data collection steps were clarified, with mock patient enrolments performed to demonstrate and confirm the understanding of trial processes by the site teams.

All training was performed online using a train-the-trainer format. The core team from FIND and the University of Oxford prepared the training materials for the trial site assessments, protocol training, data management, and methodological training for running and reporting the POC results.

A training plan was prepared for each clinical site for the trial POC testing methodology. This included preparation of training materials for good clinical laboratory practice, standard operating procedures (SOPs) for each POC test as per manufacturer guidelines, a 1-page method overview for daily use and a video film showing the correct use of each POC test together with photographing the POC test findings correctly. POC test training involved 3 days of intensive remote training for each site separately by the FIND/Oxford team, which began with desk-based training, following SOPs with questions and answers, together with a full day of practical training in the host laboratory. All of the staff using the POC tests were trained, and the trainer was able to virtually observe each trainee performing the test and assess their competency prior to beginning the study. All documentation was available on a shared folder for the sites to download, complete and keep for their records. Any new staff were trained by 1 of the staff deemed competent at the first training session.

### Layout

In the following articles we present the individual country clinical trial results, as well as the individual participant-level meta-analysis. We also present the results of qualitative research for each individual country, cross-country reflections, and outlook to the second phase of the project.

## Notes

***Author contributions***. All authors were involved in the drafting and revising of the manuscript and approved the final version for submission. Editorial assistance in the preparation of the manuscript was provided by Stuart Wakelin and funded by FIND.

***ADIP study group***. Uganda: Phyllis Awor, Deborah Ekusai-Sebatta, Heidi Hopkins, David Kaawa–Mafigiri, James Kapisi, Freddy Eric Kitutu, Elizeus Rutebemberwa, Asadu Sserwanga; Ghana: Alexander Adjei, Emmanuel Arthur, Elizabeth Awini, Rita Baiden, Vida Kukula; Clement Tetteh Narh, Gabriel Odonkor, Selase Odopey, John Williams; Burkina Faso: Adélaïde Compaoré, François Kiemde, Halidou Tinto, Daniel Valia.

***Financial support***. This part of the study was funded by the Swiss Agency for Development and Cooperation (SDC), the Federal Ministry of Economic Cooperation and Development (BMZ), and the UK DFID, now Foreign Commonwealth and Development Office (FCDO).

***Supplement sponsorship***. This article appears as part of the supplement “Using Diagnostic Tools to Support Antimicrobial Stewardship and Improve Outcome in Resource-Limited Contexts,” sponsored by FIND.
